# An all‐in‐one workflow for emergency hippocampal‐avoidance whole‐brain radiotherapy in brain metastases, with optional online adaptive extension

**DOI:** 10.1002/acm2.70405

**Published:** 2025-12-12

**Authors:** Haibo Peng, Yangang Zhou, Min Xie, Xuegui Ju, Ningyue Xu, Dong Gao, Lecheng Jia, Chunyan Dai, Huigang Tan, Tao Ren

**Affiliations:** ^1^ Department of Oncology School of Clinical Medicine, and The First Affiliated Hospital of Chengdu Medical College Chengdu Sichuan China; ^2^ Clinical Key Speciality (Oncology Department) of Sichuan Province Chengdu China; ^3^ Radiology and Therapy Clinical Medical Research Center of Sichuan Province Chengdu China; ^4^ Department of Cosmetic and Plastic Burn Surgery School of Clinical Medicine and The First Affiliated Hospital of Chengdu Medical College Chengdu China; ^5^ Department of General Medicine School of Clinical Medicine and The First Affiliated Hospital of Chengdu Medical College Chengdu China; ^6^ United Imaging Central Research Institute Co., Ltd Shanghai China; ^7^ Radiotherapy Laboratory, Shenzhen United Imaging Research Institute of Innovative Medical Equipment Shenzhen China; ^8^ Zhejiang Engineering Research Center for Innovation and Application of Intelligent Radiotherapy Technology Wenzhou China; ^9^ Shanghai United Imaging Healthcare Co., Ltd. Shanghai China; ^10^ Department of Oncology The First Affiliated Hospital of Traditional Chinese Medical of Chengdu Medical College·Xindu Hospital of Traditional Chinese Medical Chengdu China

**Keywords:** all‐in‐one radiotherapy, brain metastases, hippocampal‐avoidance whole brain radiotherapy, oncology emergencies, online adaptive radiotherapy

## Abstract

**Background:**

Brain metastases (BMs), among the most prevalent intracranial malignant tumors, frequently induce acute symptoms such as headaches and motor deficits due to elevated intracranial pressure. Although hippocampal‐avoidance whole brain radiotherapy (HA‐WBRT) has demonstrated superior preservation of cognitive function compared to standard WBRT—despite comparable intracranial progression‐free survival and overall survival outcomes—its clinical application remains restricted by the complexity and time‐intensive nature of conventional planning and delivery protocols. These limitations hinder its utility as an emergent intervention for patients requiring urgent symptom relief.

**Purpose:**

To address this challenge, we developed a novel HA‐WBRT‐specific all‐in‐one (AIO) radiotherapy integrated with online adaptive techniques, and systematically evaluated its operational feasibility, dosimetric precision, and therapeutic effectiveness for emergency HA‐WBRT in patients with BMs through preliminary clinical outcomes and related findings.

**Methods:**

The HA‐WBRT‐specific AIO workflow featured by rapid auto‐segmentation of the target and hippocampal, HA‐constrained planning using volumetric modulated arc therapy (VMAT) with three coplanar lock‐field full arcs ache ensuring a high first‐pass success rate for treatment plans, and real‐time in vivo quality assurance (QA) enabling safe beam delivery without delay was applied to the eleven emergency patients with BMs receiving HA‐WBRT during their initial treatment fraction. Rotational setup errors in subsequent fractions were documented. For these fractions, online adaptive radiotherapy (ART) was implemented using the AIO‐generated plan as the baseline. Time requirements for each workflow step and in vivo 3D gamma passing rates were analyzed to evaluate efficiency and dosimetric accuracy. Dosimetric differences between the ART‐optimized plan (ART‐Plan) and the image‐guided radiotherapy plan (IGRT‐Plan) were compared to quantify the impact of rotational deviations on dose distribution. Cognitive function (via the Mini‐Mental State Examination [MMSE]) and functional independence (via the Basic Activities of Daily Living, BADL) were assessed before and after radiotherapy.

**Results:**

The average duration of the AIO radiotherapy was 24.3 ± 0.6 min. The majority of AIO‐Plan parameters satisfied RTOG 0933 criteria. For the AIO‐Plans, in vivo 3D γ pass rates exceeded 94% (3 mm/3% gamma criteria with a 10% dose threshold). Recorded rotational errors in emergency patients exhibited irregular and substantial variations, highlighting the need for online adaptation. Notably, 94% of fractions required adaptation to meet the target coverage (TC) criteria specified by NRG CC001. All ART‐Plans demonstrated 3D γ pass rates exceeding 93% (3 mm/3% gamma criteria with a 10% dose threshold). The average duration of ART was 22.9 ± 1.6 min. Post‐radiotherapy, both MMSE and BADL scores showed measurable improvements, while acute symptoms were effectively controlled and alleviated.

**Conclusions:**

The AIO workflow for managing emergency patients with BMs is feasible and safe, facilitating prompt initiation of highly efficient radiotherapy during the first treatment session, eliminating delays. Subsequent online ART effectively corrects rotational deviations, ensuring adequate prescription dose coverage of target volumes while optimizing dose distribution to normal tissues. HA‐WBRT incorporating the AIO workflow with online adaptation demonstrates potential for timely control and alleviation of acute symptoms.

## 1 INTRODUCTION

Brain metastases (BMs), the most common intracranial tumors, occur in approximately 20% of cancer patients.^1^ Whole brain radiotherapy (WBRT) has served as the primary treatment for BMs for decades.^2^, ^3^ However, recent clinical trials, such as RTOG 0933 and NRG CC001, have demonstrated that hippocampal‐avoidance whole brain radiotherapy (HA‐WBRT) offers superior preservation of cognitive function and quality of life compared to conventional WBRT. This improvement is attributed to HA‐WBRT's targeted avoidance of the hippocampus—a critical brain structure for learning and memory—thereby shielding it from excessive radiation exposure.^4^, ^5^, ^6^

BMs are frequently associated with diverse neuropsychiatric symptoms, including elevated intracranial pressure, meningeal irritation, limb movement disorders, seizures, and cognitive decline. These manifestations represent a common oncologic emergency requiring urgent intervention. Radiotherapy has emerged as a critical salvage therapy in such emergencies. While stereotactic radiosurgery (SRS) is effective, its limited accessibility, coupled with challenges in patient tolerance of prolonged immobilization and lengthy procedural timelines, restricts its utility in acute settings. Optimal management of oncologic emergencies necessitates treatment workflows that prioritize efficiency, safety, and precise dose delivery. However, as delineated in Figure S1, the conventional workflow entails a waiting period of at least 3 days following immobilization until the first treatment. Even with the activation of the conventional emergency protocol, the pre‐treatment process still requires over 2 h, involving patient transfers between multiple clinical locations within the department, such as the CT simulator and treatment rooms. A novel “All‐in‐One” (AIO) radiotherapy platform that integrates simulation, auto‐segmentation, treatment planning, image guidance, beam delivery, and in vivo quality assurance (QA) into a single session while the patient remains on the CT‐linear accelerator (CT‐linac) couch.^7^ This streamlined approach reduces conventional multi‐day workflows to minutes, significantly enhancing patient experience and therapeutic efficiency. Although AIO's time‐sensitive design appears ideal for emergency oncology, its application in such scenarios remains unreported.

Image‐guided radiotherapy (IGRT) is widely employed for positional correction; however, standard 3‐degree‐of‐freedom (3DoF) couches cannot compensate for rotational deviations. Online adaptive radiotherapy (ART) addresses this limitation by both adapting to interfractional anatomical changes and mitigating setup inaccuracies.^8^, ^9^ Our institutional analysis of cranial treatments corroborates prior reports of clinically significant rotational deviations.^10^, ^11^, ^12^ Patients with BMs often exhibit neurological compromise, which may exacerbate postural instability during conventional fixation, further amplifying rotational inaccuracies.

We propose a novel therapeutic paradigm for emergency BMs management, combining HA‐WBRT, AIO workflow, and online ART via a CT‐linac system with diagnostic‐grade imaging. This study evaluates the feasibility, safety, and efficiency of this approach, analyzes rotational deviations’ dosimetric impact, and assesses pre‐ and post‐treatment basic activities of daily living (BADL) and short‐term neurocognitive function (NCF).

## 2 MATERIALS AND METHODS

### 2.1 Patients and equipment

Eleven patients with BMs who received emergency radiotherapy at our institution between June and December 2023 were enrolled in this study. Demographic and clinical characteristics of the cohort are summarized in Table S1. All patients exhibited acute neurological symptoms, including headache, limb impairment, and altered consciousness. BM diagnoses were confirmed via magnetic resonance imaging (MRI). This study protocol was approved by the institutional review board (IRB) of our center (No. 2023CYFYIRB‐SQ‐60), and written informed consent was obtained from all participants or their legal guardians. All procedures adhered to institutional ethical guidelines and regulatory standards. Both the online AIO radiotherapy workflow and online ART are performed using a CT‐linac called uRT‐linac 506c (United Imaging Healthcare, UIH, Shanghai, China), which combines a C‐arm linac, a kilo‐voltage 16‐slice helical fan‐beam CT (FBCT) scanner and an amorphous silicon electronic portal image device (EPID) XRD1642 (Varex Imaging Corporation, UT, USA). The integrated 16‐slice helical CT scanner enables diagnostic‐grade, high‐definition online imaging. The uRT‐linac 506c also integrates a hybrid software system consisting of a treatment planning and oncology information system (uRT‐TPOIS) and a treatment delivery system. More details concerning the uRT‐linac 506c platform have been reported.^13^ The smart planning module within the uRT‐TPOIS lays foundation for the implementation of online AIO and ART workflows. Auto‐segmentation of organs at risk (OARs) is based on a multiresolution VB‐Net convolutional neural network (CNN).^14^ This system also allows in vivo dosimetry for online QA by measuring the exit dose from the patient using the equipped EPID, which have also been demonstrated previously.^15^

### 2.2 Intelligent planning protocol

Based on the RTOG0933 study, an intelligent planning protocol was created based on a set of positioning images that had undergone HA‐WBRT.

### 2.3 Definition and delineation of targets and OARs

Auto‐segmentation OARs included the hippocampus, lenses, eyeball, optic nerves, optic chiasm, brainstem, pituitary, whole brain, spinal cord, and external. Note that the contouring of hippocampus was further confirmed on the co‐registered and fused diagnostic MRI (dMRI) axial T1‐weighted image sequence during both AIO and ART workflows, according to RTOG 0933 protocol. Expanding the hippocampus by 5 mm was defined as the area for hippocampal avoidance. The lens, eyeball, optic nerve, optic chiasm, brainstem, spinal cord, and pituitary were expanded by 3 mm to form the corresponding planning organ‐at‐risk volume. The auto‐segmented whole brain was defined as clinical target volume (CTV). To construct the planning target volume (PTV), the CTV was expanded by a 3 mm margin and the area for hippocampal avoidance was subtracted from the CTV. A template of region of interest (ROI) was developed.

### 2.4 Plan design

Volumetric‐modulated arc therapy (VMAT) with three coplanar lock‐field full arcs (bed angle: 0°, collimator angle: 90°) were adopted to design the plan of HA‐WBRT (More details in Figure S2), which was saved as the beam template. The prescription dose was 30 Gy in 10 fractions to the PTV. The optimization algorithm selected the Monte Carlo algorithm with stochastic platform optimization (SPO),^16^ and iterated 50 times. When the plan met clinical requirements, the optimization constraints were saved as a template.

### 2.5 Table of clinical goals

According to clinical requirements, clinical dosimetric goals were set for the targets and OARs, as displayed in Table S2. The ROI template, beam template, optimization constraint template, and table of clinical goals were integrated into the HA‐WBRT intelligent planning protocol.

### 2.6 Online AIO radiotherapy workflow

**FIGURE 1:**
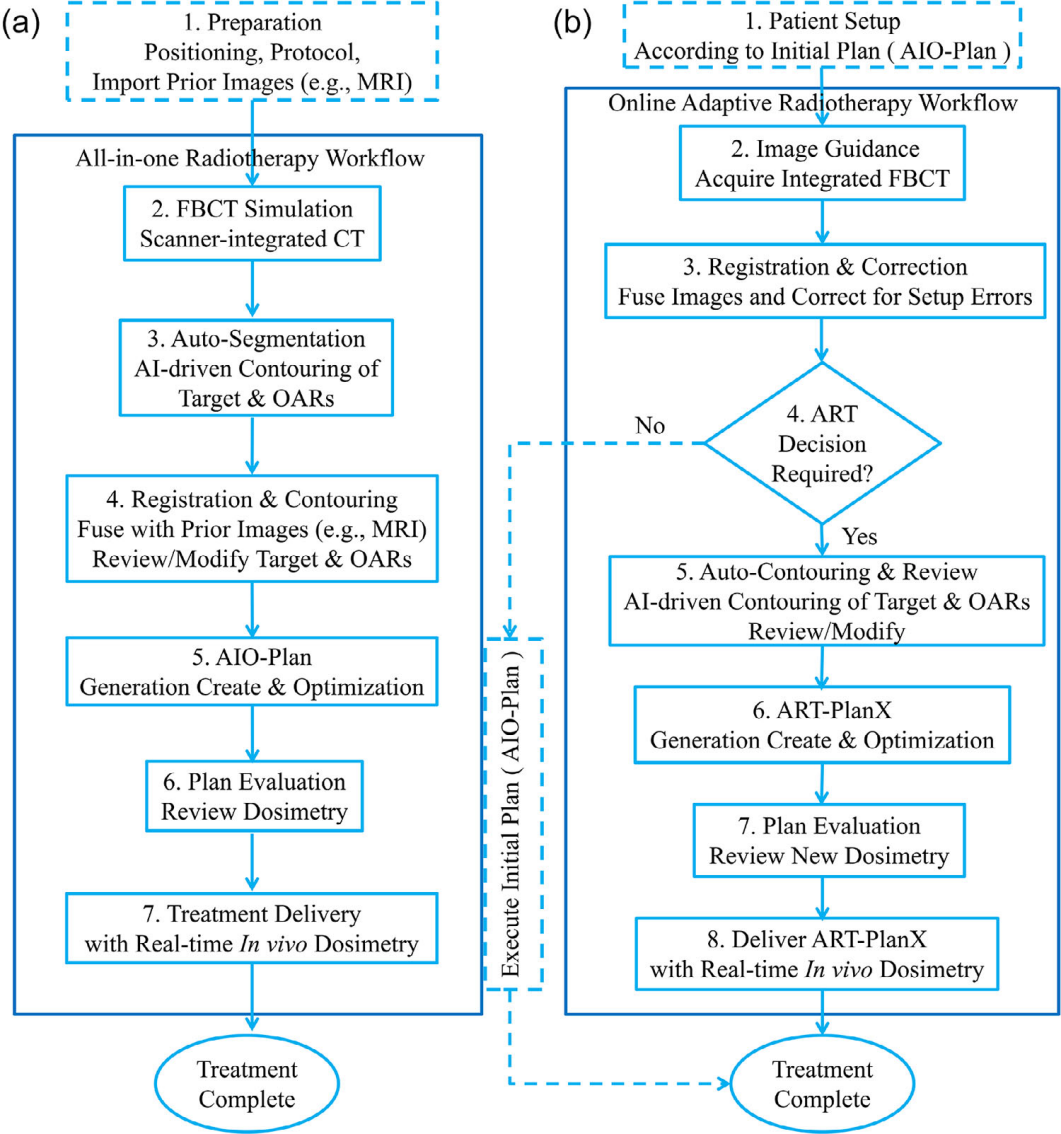
Schematic workflow for the (a) online all‐in‐one (AIO) process and (b) online adaptive radiotherapy (ART) process.

The online AIO workflow, integrated into the uRT‐TPOIS platform, consolidates essential radiotherapy procedures—from CT simulation to beam delivery—into a unified process. While key components of this workflow are automated, critical steps such as contouring and plan evaluation necessitate manual review and approval. Figure 1a shows the comprehensive breakdown of the AIO workflow:

1. Patients were positioned supine with their head centered and arms positioned laterally. A thermoplastic mask secured with a rubber headrest provided cranial immobilization.
2. Following patient registration and protocol preselection, diagnostic‐quality CT simulation was performed using the integrated uCT system (the scanning parameters are detailed in Table S3). Acquired images were automatically transferred in DICOM format to the treatment planning system (TPS) via the uRT‐TPOIS server.
3. The TPS autonomously generated contours for OARs and target volumes based on the preset protocol. On‐site, radiation oncologists verified and manually adjusted these structures as needed, with hippocampal delineation requiring cross‐referencing to axial T1‐weighted dMRI sequences.
4. The TPS automatically created a plan (AIO‐Plan) based on the intelligent planning protocol and calculated and optimized the dose according to the corresponding priority of the table of clinical goals. This step could be performed by manually modifying the constraints, as appropriate.
5. The optimized plan underwent a multidisciplinary review, conducted on‐site by radiation oncologists and medical physicists, focusing on compliance with clinical goals. Following approval, an in vivo dose verification document was generated and transferred to the treatment delivery system (TDS).
6. During first‐fraction irradiation, EPID measurements enabled real‐time dose verification. Predicted doses (calculated via fast Monte Carlo algorithms) were compared to EPID‐acquired doses using 3 mm/3% gamma criteria with a 10% dose threshold.
7. EPID measurements were inversely reconstructed (global gamma criteria: 3 mm/3%; dose threshold: 10%; uncertainty limit: 0.5%) to generate a 3D delivered dose map for daily treatment validation.
8. Time required for each step of the AIO workflow was recorded.

### 2.7 Online ART workflow

The online ART workflow is similar to that of AIO. Figure 1b illustrates the detail of online ART workflow:

1. From the second fraction onward, the AIO‐Plan was invoked each time and placed according to its parameters.
2. The integrated CT was used to perform image guidance according to the positioning scanning parameters (more details in Table S3).
3. The acquired image was co‐registered with the reference positioning image to calculate six‐dimensional (6D) deviations (X, Y, Z, Roll, Pitch, and Yaw). Translational errors (X, Y, and Z) were corrected via 3D couch shifts, while rotational deviations (Roll, Pitch, and Yaw) were documented.
4. The TPS automatically segmented targets and OARs, excluding the hippocampus, to generate adaptive structures on the current image. Hippocampal contours were derived via rigid registration, resulting in minimal manual adjustments. Final target and OAR delineations were verified by both radiation oncologists and medical physicists.
5. An online adaptive plan (ART‐Planx) was automatically generated and optimized. The adaptive algorithm incorporated dose predictions and the original plan's clinical goals as inputs, accounting for patient‐specific anatomical geometry. Priorities outlined in the clinical goal sheet provided explicit optimization criteria, enabling derivation of dosimetric parameters for the adaptive plan.
6. Post‐optimization, the ART‐Plan was evaluated by clinicians, either on‐site or online, focusing on compliance with clinical goal criteria. Plans meeting requirements were approved exclusively for the current fraction.
7. The approved ART‐Plan was synchronized with the TDA. During delivery, in vivo dose verification via EPID provided real‐time 2D γ passing rates. Post‐treatment, 3D dose reconstruction and γ analysis were performed.
8. Time intervals for each workflow step were recorded.
9. Following ART‐Plan delivery, the AIO‐Plan was mapped to the corrected image (with applied translational shifts) to generate an image‐guided radiotherapy plan (IGRT‐Planx).
10. Dosimetric parameters of ART‐Plan and IGRT‐Plan were compared. For target, the target coverage (TC) of prescription dose; maximum dose (D_max_); dose of 2% (D_2_), 98% (D₉₈), and 50% (D_50_) volume of PTV, conformity index (CI) and homogeneity index (HI) were compared. For OARs, D_max_ of hippocampus, lenses, optic nerves, optic chiasm, brainstem were compared. The mean dose of hippocampus was also compared. Where CI = (V_T_, ref)^2^/(V_T_ · V_ref_), V_T_, ref is volume of PTV receiving prescription dose or more (cm^3^), V_T_ is PTV (cm^3^), V_ref_ is volume of reference isodose line (cm^3^); HI = (D_2_–D₉₈)/D_50_; V₁₀₀% and V_50_% are the volume of prescription isodose and 50% prescription isodose curves.

### 2.8 Assessment of NCF and ADL

The Mini‐Mental State Examination (MMSE) and the BADL were adopted to evaluate the short‐term NCF and the ADL.^17^, ^18^ MMSE and BADL were tested for three times: pre‐treatment, post‐treatment, and 3 months after treatment.

### 2.9 Statistical analysis

Statistical Product and Service Solutions (IBM SPSS, version 20.0; New York, NY, USA) were used to perform statistical analysis among results of dosimetric parameters obtained with ART‐Plan and IGRT‐Plan, represented by mean ± standard deviation (SD). The Shapiro‒Wilk method was used to test the normality of the data. The paired test was performed for data lines with a normal distribution. Differences were regarded as statistically significant if *p* < 0.05.

## 3 RESULTS

### 3.1 AIO workflow evaluation

**FIGURE 2:**
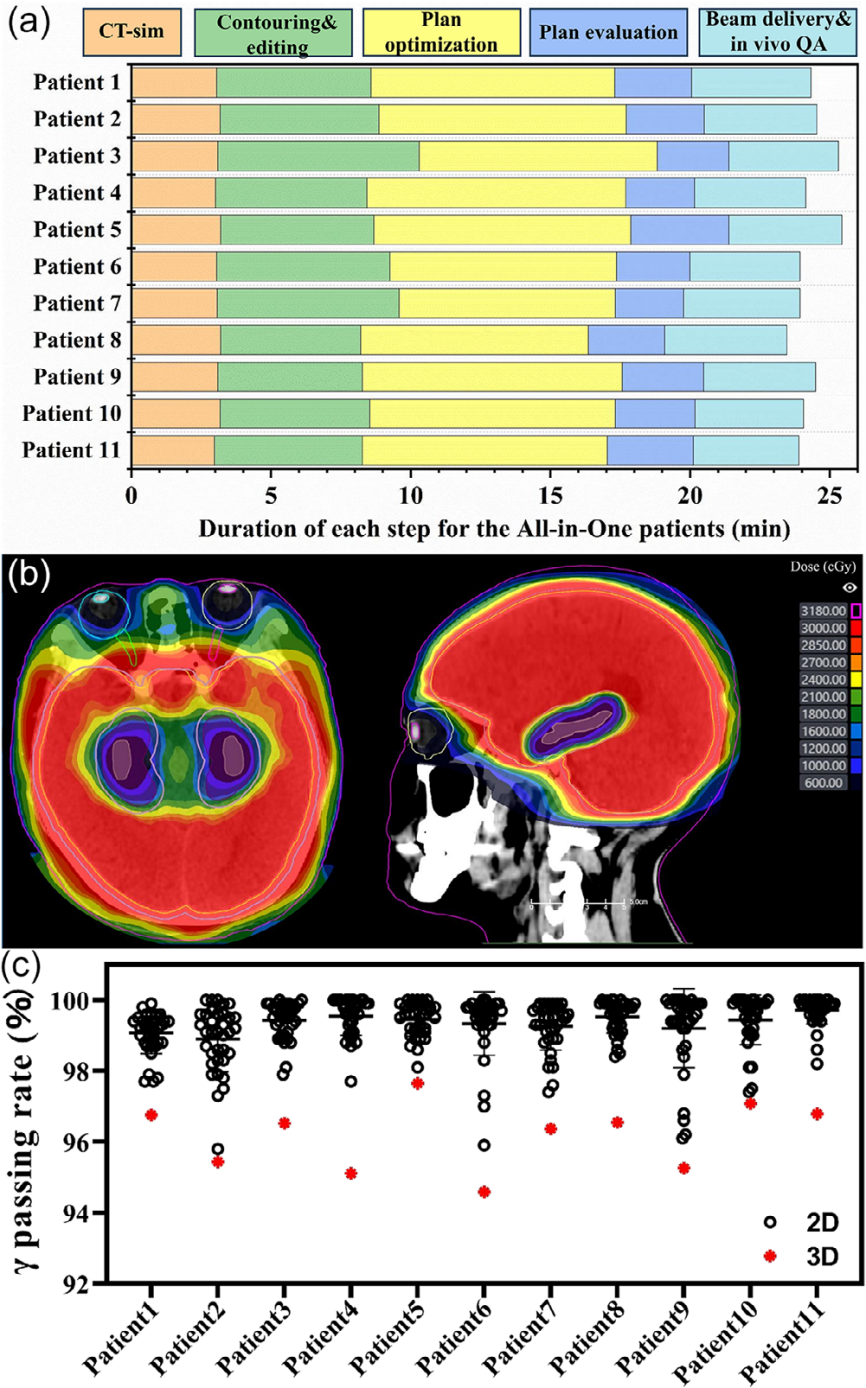
(a) Time required in each phase of the all‐in‐one (AIO) workflow for the emergency patients. (b) Dose distributions with isodose lines for a representative patient. (c) γ passing rates of in vivo 2D (black circle) and 3D reconstruction (red star) for the AIO patients.

All the 11 patients successfully completed the online AIO radiotherapy for their first treatments. Figure 2a displays the duration for the AIO process as well as times of each step for the patients. Among 11 patients, the total time for the AIO workflow was 24.3 ± 0.6 min. All patients completed the full workflow within 30 min. Among all steps, plan optimization required an average time determined to be 8.7 ± 0.5 min (range: 7.7 to 9.3 min). Auto‐segmentation and contour adjustment took up an average of time measured to be 5.7 ± 0.7 min (range: 5.0 to 6.5 min). It should be noted that the CTV for HA‐WBRT was determined to be the whole brain volume, and the auto‐segmented “target” and OARs including lens, eyes, optic chiasm, optic nerve were clinically accepted for all cases without any modification. The consumption of time for this phase was mainly due to the modification of hippocampus, whose contouring, according to the RTOG 0933 protocol, needed to refer to the MRI axial T1‐weighted image sequence.

Dose normalization was performed for all the AIO‐Plans to ensure at least 95% of PTV receiving prescription dose. All other parameters of AIO‐Plans met RTOG 0933 criteria except D_98%_ of patient 5 slightly < 25 Gy. While the D_max_ objective per NRG CC001 was not met for optic structures for all patients, they were quite close and reduced by 15%–20% below the constraints specified by RTOG 0933 (Table 1). Figure 2b shows the dose distribution with color wash comparison of a representative patient. The developed VMAT with three coplanar lock‐field full arcs could not only meet clinical constraints of HA‐WBRT plan, but also shorten delivery time to 4.0 ± 0.2 min.

**TABLE 1:** Dose parameters for the all‐in‐one (AIO) plans of the patients.

ROI	NRG CC001	RTOG 0933	1	2	3	4	5	6	7	8	9	10	11
PTV	V_30Gy_ ≥ 95%	V_30Gy_ ≥ 90%	95	95	95	95	95	95	95	95	95	95	95
	D_2%_ ≤ 37.5 Gy	D_2%_ ≤ 37.5 Gy	34.58	34.12	34.37	34.18	34.59	34.07	33.26	33.06	34.49	33.46	35.77
	D_98%_ ≥ 25 Gy	D_98%_ ≥ 25 Gy	25.17	25.72	25.68	25.71	24.81	25.01	26.43	25.69	25.37	25.53	25.98
Hippocampi	D_100%_ ≤ 9 Gy	D_100%_ ≤ 9 Gy	8.52	8.11	8.18	8.23	8.50	8.55	7.69	8.30	8.22	8.27	8.11
	D_max_ ≤ 16 Gy	D_max_ ≤ 16 Gy	10.74	14.62	12.56	11.02	10.52	10.49	9.11	11.40	11.52	10.74	12.29
Optic chiasm	D_max0.03cc_ ≤ 30 Gy	D_max_ ≤ 37.5 Gy	32.44	31.78	32.29	32.11	32.53	31.88	31.23	30.78	32.28	31.39	32.67
OpticNrv_L	D_max0.03cc_ ≤ 30 Gy	D_max_ ≤ 37.5 Gy	30.78	28.27	31.26	30.24	31.63	30.71	28.65	29.75	30.84	29.81	29.91
OpticNrv_R	D_max0.03cc_ ≤ 30 Gy	D_max_ ≤ 37.5 Gy	31.35	30.11	31.21	30.60	31.76	31.26	29.04	29.25	31.16	30.18	26.91

Abbreviations: PTV, planning target volume; ROI, region‐of‐interest.

The safety of the AIO workflow was confirmed by the in vivo QA during beam delivery and 3D dose reconstruction analysis. The mean γ passing rate for the 11 AIO patients is presented in Figure 2c. Almost all the AIO patients showed a γ passing rate better than 96% at all the checkpoints (3%/3 mm, 10% threshold). The mean 3D γ passing rates of each case were all higher than 94%.

### 3.2 Difference in head position

**FIGURE 3:**
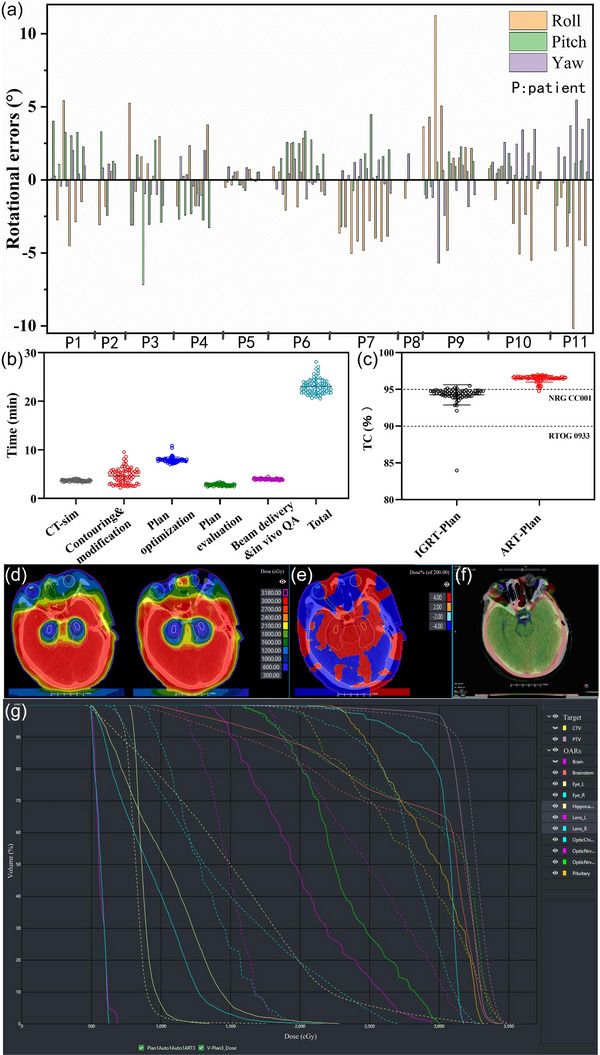
(a) Rotational errors of roll, pitch and yaw for online adaptive radiotherapy (ART). (b) Time required in each phase of the ART workflow for the emergency patients. (c) Distribution of target coverage (TC) for the image‐guided radiotherapy plan (IGRT‐Plan) and ART‐optimized plan (ART‐Plan). (d) Dose distribution for ART‐Plan (left) and IGRT‐Plan (right) of a representative patient. (e) Dose difference between the representative patient' ART‐Plan and IGRT‐Plan. (f) Representative patient' rotational errors after registration (grey indicates ART fraction; color indicates all‐in‐one [AIO] fraction). (g) Dose volume histograms (DVH) of between the representative patient' ART‐Plan and IGRT‐Plan.

For the overall 11 patients, both the translational errors in left‐right (LR), superior‐inferior (SI), and anterior‐posterior (AP) directions and rotational errors in pitch, roll, and yaw directions were measured. Translational errors for LR, SI, and AP directions were calculated to be 0.12 ± 0.10 cm, 0.19 ± 0.14 cm, and 0.06 ± 0.06 cm, respectively. In contrast, the rotational errors for roll, pitch, and yaw corrections that cannot be corrected by 3DoF couches were determined to be 2.61 ± 2.17°, 1.67 ± 1.32°, and 1.35 ± 1.19°, respectively. Figure 3a illustrates the distribution of rotational errors for these patients. It can be clearly seen that rotational errors tend to be more fluctuant throughout the entire course of radiotherapy. Among these rotational errors, these patients seemed to be more susceptible to the roll error.

### 3.3 Online ART

Figure 3b illustrates the duration for the online ART process and time expended in each step of the ART workflow. Among the 11 patients, the online ART workflow took a total time of 22.9 ± 1.6 min from CT simulation to beam delivery. Contour modification (4.4 ± 1.4 min; range: 2.2 to 8.6 min) and plan optimization (7.9 ± 0.6 min; range: 7.0 to 10.8 min) were the longest steps for ART. In terms of target dose distribution, although all IGRT‐Plan met TC requirement of RTOG 0933 (V_30Gy_ ≥ 90%), 94% of them required adaptation to meet that of NRG CC001 (V_30Gy_ ≥ 95%) (Figure 3c). The mean TC of PTV for ART‐Plan and IGRT‐Plan were measured to be 96.4% and 94.2%, respectively, increased by an average of 2.2% after adaption, with statistically significant difference (*p* < 0.05). D_max_, D_98%_, D_50%_, D_2%_, HI, and CI of ART‐Plan were superior to those of IGRT‐Plan, all these target parameters showed statistically significant differences. It is worth noting that D_98%_ of ART‐Plan (27.8 ± 0.69 Gy) met the requirements of NRG CC001 and RTOG 0933, while that of IGRT‐Plan (24.6 ± 0.97 Gy) failed (Table 2). An illustrative patient' dose distribution and dose volume histograms (DVH) between ART‐Plan and IGRT‐Plan are displayed in Figure 3d,e, and g. Apparent dosimetric advantages can be observed after online adaption, which should be closely related to the correction of rotational errors after registration (Figure 3f).

**TABLE 2:** Comparison of dosimetric parameters of various planning target volume (PTV) and organs at risk (OARs).

Parameters		IGRT‐Plan	ART‐Plan	*p*‐value
PTV	TC (%)	94.25 ± 1.37	96.45 ± 0.45	<0.05
	D_max_ (Gy)	35.39 ± 0.92	34.85 ± 0.60	<0.05
	D_2%_(Gy)	34.18 ± 0.76	33.49 ± 0.44	<0.05
	D_50%_(Gy)	32.68 ± 0.58	32.11 ± 0.32	<0.05
	D_98%_(Gy)	24.58 ± 0.98	27.83 ± 0.69	<0.05
	CI	0.82 ± 0.04	0.89 ± 0.03	<0.05
	HI	0.29 ± 0.04	0.18 ± 0.02	<0.05
Hippocampus	D_max_ (Gy)	13.79 ± 3.47	11.70 ± 0.84	<0.05
	D_mean_ (Gy)	8.51 ± 0.49	8.98 ± 0.43	<0.05
Lens_L	D_max_ (Gy)	7.65 ± 2.93	5.43 ± 0.69	<0.05
Lens_R	D_max_ (Gy)	7.26 ± 2.62	5.25 ± 0.46	<0.05
Optic chiasm	D_max_ (Gy)	32.31 ± 0.96	31.52 ± 0.40	<0.05
OpticNrv_L	D_max_ (Gy)	30.93 ± 0.88	28.83 ± 1.60	<0.05
OpticNrv_R	D_max_ (Gy)	30.49 ± 2.02	29.17 ± 1.44	<0.05
Brainstem	D_max_ (Gy)	34.84 ± 0.75	34.36 ± 0.63	<0.05

Abbreviations: ART‐Plan, ART‐optimized plan; IGRT‐Plan, the image‐guided radiotherapy plan; TC, target coverage.

Besides target dose distribution, OARs also benefit from adaptation. As shown in Table 2, both ART‐Plan and IGRT‐Plan met the RTOG 0933 protocol dose compliance criteria for hippocampal sparing. Hippocampal D_max_ of ART‐Plan was superior to that of IGRT‐Plan, with statistically significant difference (*p* < 0.05). Apparent improvement was observed for lens. The D_max_ of left lens and right lens decreased by 28.9% and 28.7%, respectively, after adaptation, with statistically significant difference. In addition, compared to IGRT‐Plan, ART‐Plan yielded lower D_max_ to optic chiasm, optic nerves and brainstem. Note that D_max_ of optic nerves (left: 28.8 ± 1.60 Gy; right: 29.2 ± 1.44 Gy) further met constraint of NRG CC001 (D_max_ ≤ 30 Gy) after adaptation.

**FIGURE 4:**
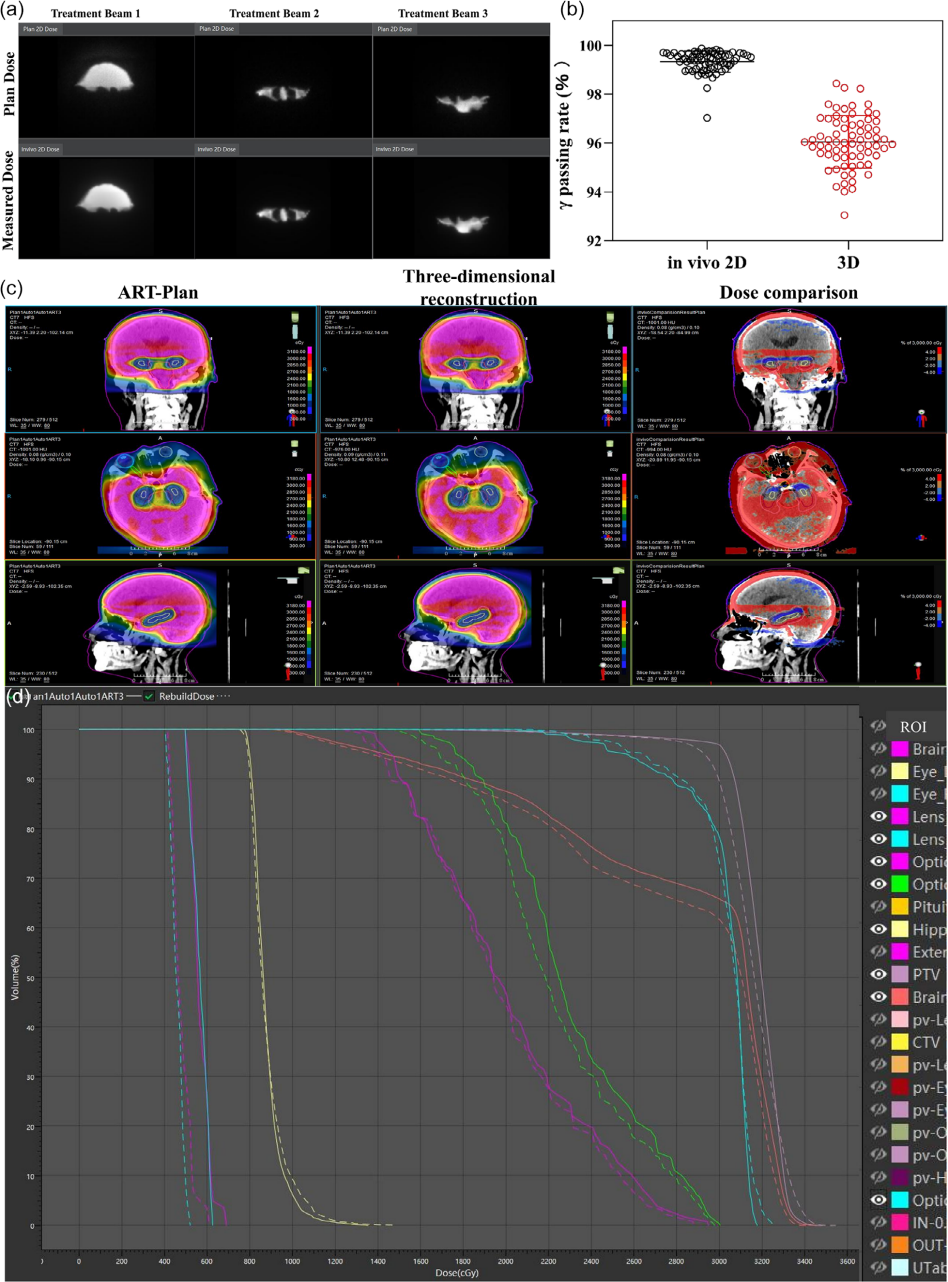
(a) 2D in vivo dose monitoring for three treatment beams. (b) γ passing rates of in vivo 2D and 3D reconstruction for adaptive radiotherapy (ART) fractions. (c) Dose distribution results between the representative patient' ART‐Plan and 3D reconstruction. (d) Dose volume histograms (DVH) of between the representative patient' ART‐Plan and 3D reconstruction.

In vivo QA during beam delivery and 3D dose reconstruction analysis were used to ensure safety of the ART workflow. As shown in Figure 4a–b, all the patients showed a mean γ passing rate of > 97% (3%/3 mm, 10% threshold) for ART fractions. 3D reconstruction was able to reflect the dose that the patient actually received during treatment. The mean γ passing rates for all the ART fractions of these patients were all higher than 93% (Figure 4b). Figure 4c and d displays the dose distribution and DVH between an ART plan and the corresponding 3D reconstruction, for the patient illustrated in Figure 3, suggesting the accurate dose delivery for the online ART.

### 3.4 Clinical evaluation

Table S4 summarized the MMSE and BADL scores for 11 patients at three timepoints. Patient 2 discontinued radiotherapy after two fractions due to personal circumstances. Four patients died within 3 months post‐treatment, and one patient died within 6 months; five patients remained alive at the time of reporting.

Among these patients (excluding Patient 2), seven patients exhibited improved MMSE scores immediately following radiotherapy, while three patients‐maintained pre‐treatment levels. Three months after radiotherapy, MMSE scores among survivors showed minimal deviation from post‐radiotherapy values. In contrast, nine patients demonstrated significant improvement in BADL scores post‐treatment, with only Patient 3 retaining pre‐radiotherapy BADL scores. Notably, Patient 3 presented a baseline BADL score of 5 points, indicative of profound functional impairment. By the 3‐month follow‐up, BADL scores among survivors fluctuated marginally compared to immediate post‐treatment values.

## 4 DISCUSSION

RTOG 0933 and NRG Oncology CC001 have demonstrated that HA‐WBRT effectively preserves NCF by minimizing hippocampal dose while ensuring coverage of known and potential BMs during treatment.^5^, ^6^ However, conventional HA‐WBRT workflows are labor‐intensive, time‐consuming, and reliant on multi‐device, multi‐location processes, which compromise timeliness—a critical consideration for BM patients with NCF. Furthermore, the logistical complexity of this approach elevates the risk of procedural errors, potentially undermining dose delivery accuracy.

The integration of AIO radiotherapy and online ART, based on HA‐WBRT offers a novel solution to the aforementioned challenges. First, AIO radiotherapy enhances treatment timeliness and precision for emergency cases. In conventional radiotherapy workflow, patients first undergo a simulation CT scan, after which physicians contour the target volumes and physicists complete the treatment plan, which is a multi‐step process, and patients usually suffer from days‐long delays before initial treatment—a process that exacerbates physical and psychological burdens for emergency patients and their families. In contrast, this study employed AIO radiotherapy to eliminate multi‐room transfers and streamline workflows by integrating simulation, contouring, planning, and delivery while patients remained on the treatment couch. The total AIO workflow duration, from CT simulation to beam delivery, was 24.3 ± 0.6 min. In order to realize a rapid HA‐WBRT‐specific AIO workflow, two technically demanding steps critical to AIO implementation are auto‐segmentation of hippocampal and HA‐WBRT plan design. While the RTOG 0933 protocol mandates hippocampus delineation on MRI axial T1‐weighted sequences,^19^ CT‐based auto‐segmentation of the hippocampus has demonstrated clinical feasibility. In this workflow, all OARs were auto‐segmented, with hippocampal contours subsequently verified and adjusted against dMRI T1‐weighted reference images, accounting for the majority of contouring time. However, it should be noted that current auto‐segmentation model for hippocampal showed certain degree of deviation compared to MR‐based contouring (Figure S3), which cause the main consumption of modification time. A more accurate segmentation model for hippocampal can help further improve workflow efficiency in the future.

Rapid and accurate HA‐WBRT plan design remains a challenge for online AIO systems. The RTOG 0933 guidelines recommend helical tomotherapy (Tomo) or nine‐field non‐coplanar IMRT for HA‐WBRT. While Tomo achieves optimal hotspot control and TC, its limited availability in most centers and prolonged delivery times (> 20 min) hinder widespread adoption.^20^ Non‐coplanar arc techniques have shown comparable TC and improved lens/optic nerve sparing.^21^ Recent advancements in coplanar partial‐field full arcs enable HA‐WBRT delivery in ≤ 5 min with equivalent TC and OAR protection.^22^ For this study, three unique coplanar lock‐field full arcs were developed to prioritize timeliness for emergency cases. The split‐field design minimized multileaf collimator (MLC) travel distances, enhancing modulation capacity and hippocampal dose reduction. During AIO workflows, plans were auto‐generated via a predefined smart protocol. Optimization required 8.7 ± 0.5 min (range: 7.7–9.3), constituting the workflow's longest phase yet remaining competitive with alternative technologies. Coplanar beam delivery time was 4.0 ± 0.2 min, significantly shorter than Tomo, non‐coplanar VMAT, or IMRT.

Because the skull is approximately elliptical and connected to the movable cervical vertebrae, asymptomatic cranial radiotherapy patients may experience significant setup deviations during the treatment fractions when fixed with standard rubber pillows combined with thermoplastic mask.^23^, ^24^, ^25^ For emergency patients with BMs, reduced patient compliance further complicates setup error control. While translational errors can be corrected via treatment couch adjustments, rotational errors cannot be addressed by conventional 3DoF couches. Current mitigation strategies—repeated setup processes and iterative image guidance—are time‐consuming and inaccurate. To ensure dosimetric precision and mitigate rotational error impacts, an online ART workflow integrated into a CT‐linac system was implemented for subsequent treatment fractions. This approach‐maintained target dose distributions and OARs constraints more effectively than IGRT‐Plan, potentially enhancing clinical efficacy. This approach‐maintained target dose distributions and OARs constraints more effectively than IGRT‐Plan. As shown in Table 2, all dosimetric parameters demonstrated statistically significant improvements. Furthermore, the ART‐Plan demonstrated better compliance with the clinical objectives outlined in Table S2, suggesting a potential enhancement in clinical efficacy. The online ART workflow employed a dose prediction‐based adaptive optimization algorithm,^26^ which balanced individualized patient needs and adaptive plan's first‐pass rate. In the adaptive optimization algorithm, the inputs consist of the original plan's dose distribution and its associated clinical goal sheet. This goal sheet includes a prioritized list of objectives that specify the required prescription dose coverage for the PTV and the dosimetric constraints for OARs.

Leveraging these inputs and accounting for anatomical variations, the algorithm infers appropriate dosimetric parameters for the adaptive plan. When clinical goal priorities remain consistent across cases, a standardized goal sheet can be applied to similar treatment plans. In scenarios where substantial anatomical changes create conflicts between TC and OAR sparing, the algorithm prioritizes fulfillment of high‐priority goals first and then optimizes as much as possible toward achieving lower‐priority objectives. By utilizing these strategies, adaptative plans that not only take individualized differences of patients into account, but also meet the clinical objectives can be generated automatically, greatly shortening the time of plan design and improving the homogeneity of adaptative plan.

During the processes of online AIO and ART, these patients remained on the treatment couch until the end of treatment, thus pre‐treatment plan validation is not feasible. In vivo QA based on integrated EPID of CT‐linac was adopted to monitor real‐time 2D γ passing rate which was checked every 30° during beam delivery. The standard of γ passing rate was set as 3%/3 mm/10% threshold, and a warning would be issued when the γ passing rate was lower than 88%. For all the treatment fractions in this study, no warning prompts occurred, and the lowest 2D γ passing rate was higher than 97%. The collected 2D dose information was further utilized to perform 3D dose reconstruction and γ analysis (3%/3 mm, 10% threshold), aiming at comprehensively verifying the accuracy of plan implementation. During plan delivery, key delivery parameters, such as MLC positions, collimator angles, and the machine's actual beam output, were encoded in the in vivo 2D measurement images acquired in real time. The in vivo 3D dose reconstruction was then derived from these treatment‐acquired 2D images. Specifically, fluence maps extracted from the 2D data were fused with the patient's CT anatomy to reconstruct the delivered 3D dose distribution. This reconstruction was performed using the fast Monte Carlo Dose Algorithm developed by United Imaging Healthcare, a Monte Carlo dosimetry algorithm, which enabled to accurately calculate the patient's in vivo 3D reconstruction dose field.^13^, ^27^ 3D γ passing rate in this study was all higher than 93%, further confirming safety, reliability and accuracy for the AIO and ART workflows, laying the foundation for clinical benefits of patients.

For patients with BMs, especially this part with advanced or terminal‐stage, maintaining NCF and ADL are at least as important as prolonging survival. Therefore, measurements of NCF and ADL have become important indexes for these patients. In our study, NCF and ADL of these patients were tested using MMSE and BADL, at three time points: pre‐treatment, post‐treatment, and 3 months after treatment. The results demonstrated that the acute symptoms in patients who completed the radiotherapy regimen were effectively controlled and alleviated. Following radiotherapy, both MMSE and BADL scores remained stable or even showed improvement in some cases. This outcome is consistent with previous reports in the literature, and may also be attributed to the relatively low baseline values observed in this study population, which presented with acute symptoms.^5^, ^6^, ^28^ Given the limited sample size and non‐randomized design of this study, further validation through larger prospective investigations is warranted.

It should be noted that in this study, we focused on the comprehensive radiotherapy workflow management for patients with BMs in emergency. During the whole radiotherapy process, the AIO workflow was proposed for the patient's first radiotherapy, which enabled rapid treatment and promptly alleviated symptoms for these patients. The subsequent fractions were treated using online ART workflow, aiming at deal with potential rotational errors for these patients which could not be corrected by 3DoF treatment couch. For emergency patients with brain metastases, the ability to maintain immobilization may be compromised to some extent, and the rotational deviations cannot be ignored in the absence of 6DoF treatment couch.^29^ Therefore, we proposed that the ART workflow was a meaningful and valuable alternative for subsequent treatment fractions for these emergency patients, and was indispensable for the comprehensive radiotherapy workflow management for patients with BMs in emergency.

This study has several limitations. First, the online AIO workflow necessitates substantial manual correction of auto‐segmented hippocampal contours, and the current HA‐WBRT plan remains dependent on a predefined template. Enhancing the auto‐segmentation model for the hippocampus and developing an automated HA‐WBRT planning system could streamline these steps, reducing contouring and planning durations to improve workflow efficiency. Second, the process requires continuous on‐site or online oversight by radiation oncologists and medical physicists to ensure safety. Optimizing patient positioning protocols and enhancing setup reproducibility may reduce the frequency of online adaptive interventions. Finally, this study suffered from limited patients and was unable to analyze clinical benefits comprehensively.

## 5 CONCLUSIONS

The AIO radiotherapy enabled patients with acute BMs to complete the first radiotherapy in a single space within a short time, and the whole process was efficient and safe. For subsequent fractions, the online ART workflow eliminated positional inaccuracies, particularly uncorrectable rotational deviations inherent to 3DoF couches. This approach further ensured the prescription dose coverage of the target and the limitation of the OARs, demonstrated superior dosimetric performance compared to IGRT. AIO combined with online ART may help to rapidly control and alleviate the symptoms of patients with acute BMs and ensure that the hippocampal dose parameters within safe thresholds, which is expected to enhance the quality of life for patients and create opportunities to receive subsequent systemic therapies.

## AUTHOR CONTRIBUTIONS

Haibo Peng, Yangang Zhou, and Tao Ren contributed substantially to the conception and design of the work. Min Xie, Xuegui Ju, Ningyue Xu, Dong Gao, Lecheng Jia, Chunyan Dai, and Huigang Tan participated in the acquisition, analysis, and interpretation of data for the work. Haibo Peng, Tao Ren, and Dong Gao drafted the work and revised it critically for important intellectual content. All authors gave final approval of the version to be submitted for review and agreed to be accountable for all aspects of the work in ensuring that questions related to the accuracy or integrity of any part of the work are appropriately investigated and resolved.

## CONFLICT OF INTEREST STATEMENT

The authors declare no conflicts of interest.

## Supporting information



Supporting Information

## Data Availability

Data will be made available on request.
