# Supplementary material for: An all‐in‐one workflow for emergency hippocampal‐avoidance whole‐brain radiotherapy in brain metastases, with optional online adaptive extension
Source: J Appl Clin Med Phys. 2025 Dec 12;26(12):e70405. doi: 10.1002/acm2.70405 (PMC12699192; doi:10.1002/acm2.70405)
Supplement: Supplementary file 1 — Supporting Information [file ACM2-26-e70405-s001.docx]

**Supporting Information**

**
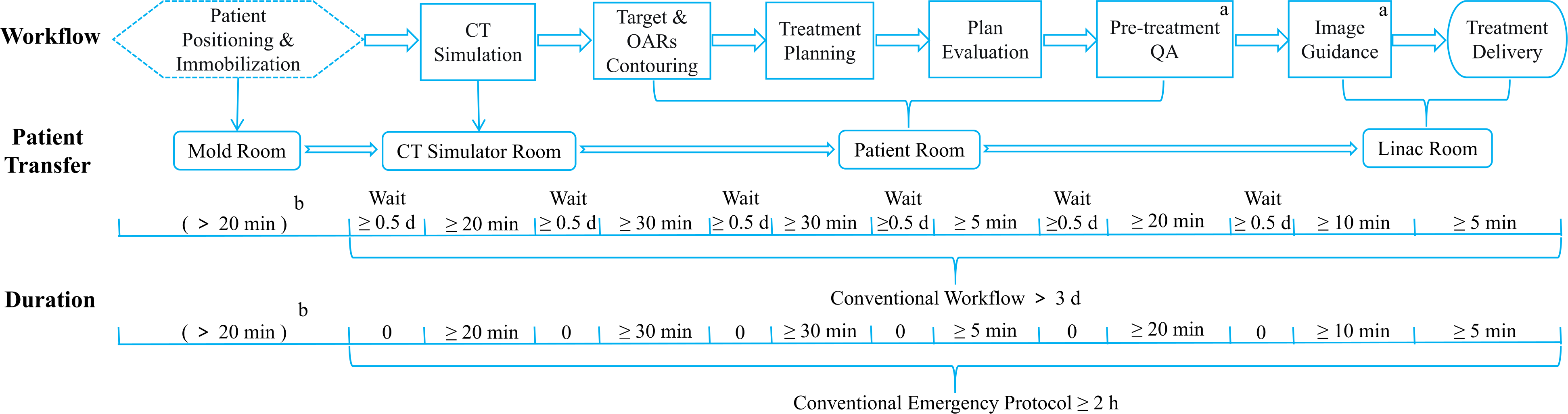
**

Note: **a.** Additional steps not present in the all-in-one radiotherapy workflow. **b**. Time for immobilization not accounted for in the conventional radiotherapy workflows total, as the all-in-one radiotherapy workflow initiates from simulation.

Abbreviations: CT, computed tomography; OARs, organs at risk; QA, quality assurance.

**FIGURE S1** Conventional HA-WBRT workflow and conventional emergency protocol with their respective time consumption, based on the clinical practice in our department.

**TABLE S1** Characteristics of patients.

| Case | Sex | Age | Primary tumor | Site of BMs | Number of BMs | Maximum diameter of BMs (cm) | Acute symptoms | Other metastases |
| --- | --- | --- | --- | --- | --- | --- | --- | --- |
| 1 | Male | 71 | LUSC | Frontal lobe  Parietal lobe  Occipital lobe | ≥3 | ＜3 | Headache; Left limb weakness | N |
| 2 | Male | 73 | SUS | Parietal lobe  Occipital lobe | ≥3 | ＜3 | Headache | Y |
| 3 | Female | 52 | LUAD | Frontal lobe  Parietal lobe | ≥3 | ＜3 | Headache; Limbs weakness | Y |
| 4 | Male | 83 | SCLC | Frontal lobe  Parietal lobe | ＜3 | ≥3 | Headache; Nausea; Vomiting; Difficulty breathing | Y |
| 5 | Male | 64 | LUAD | Parietal lobe | ＜3 | ≥3 | Limbs weakness;  Nausea; Vomiting | N |
| 6 | Male | 48 | LUAD | Occipital lobe  Cerebellum  Meninx | ≥3 | ＜3 | Headache; Nausea; Vomiting | Y |
| 7 | Female | 77 | LUAD | Frontal lobe  Parietal lobe  Cerebellum | ≥3 | ＜3 | Headache;  Limbs weakness;  Visual rotation | N |
| 8 | Male | 78 | ESCC | Frontal lobe  Parietal lobe  Occipital lobe  Temporal lobe | ≥3 | ＜3 | Headache;  Limbs weakness | Y |
| 9 | Female | 47 | LUAD | Frontal lobe  Parietal lobe  Temporal lobe  Meninx | ≥3 | ＜3 | Headache; Nausea; Vomiting;  Blurred vision | N |
| 10 | Male | 65 | LUAD | Occipital lobe  Parietal lobe  Temporal lobe  Meninx | ≥3 | ＜3 | Headache | N |
| 11 | Male | 69 | Hidradenocarcinoma | Frontal lobe  Parietal lobe | ≥3 | ＜3 | Headache | Y |

Abbreviations: BMs, brain metastases; SUS, spindle undifferentiated sarcoma; LUSC, lung squamous cell carcinoma; LUAD, lung adenocarcinoma; SCLC, small cell lung cancer; ESCC, esophageal squamous cell carcinoma.

**
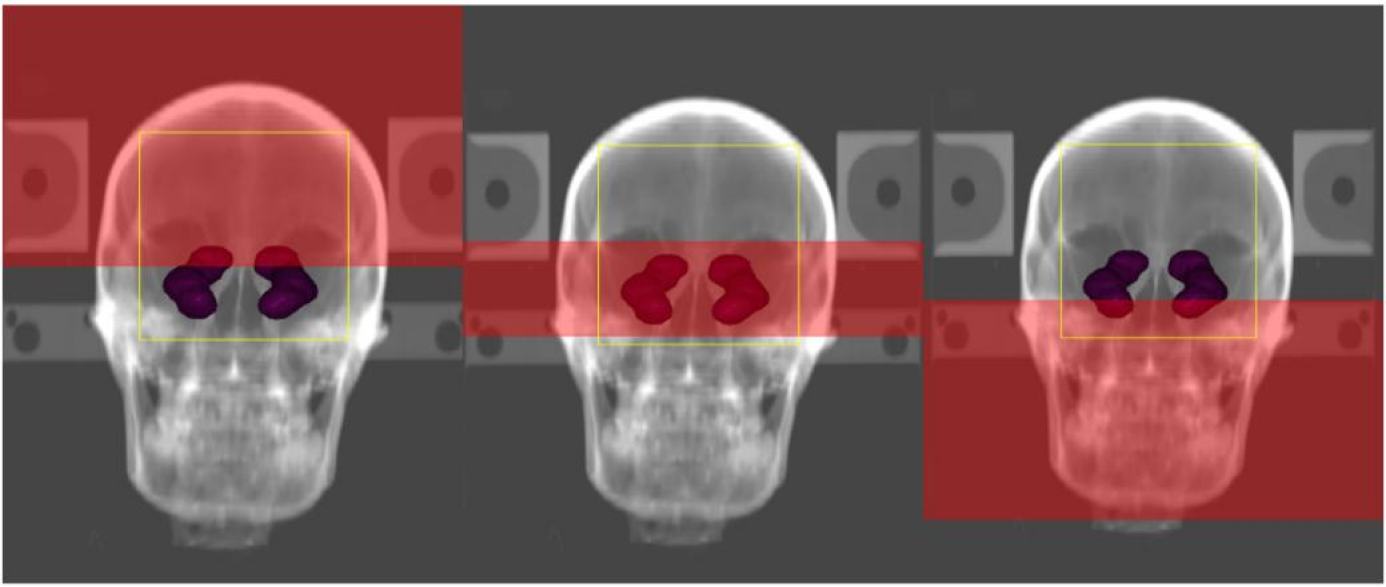
**

**FIGURE S2** Scheme of three coplanar lock-field full arcs for HA-WBRT. The lower edge of the locking field for the first arc is located 1 cm below the top layer of the hippocampus. For the second arc, the upper edge and the lower edge of the locking field are located 1 cm above the bottom layer of the hippocampus and below the top layer of the hippocampus, respectively. For the third arc, the upper edge of the lock field is located 1 cm above the bottom layer of the hippocampus. These visible high-Z features correspond to the indexing bars and adapter components of the immobilization baseplate.

**TABLE S2** Clinical goals for HA-WBRT plan.

| ROI | Clinical goal |
| --- | --- |
| PTV | D_95%_≥3000cGy |
|  | D_max_＜3750cGy |
|  | D_98%_≥2500cGy |
| Hippocampus | Dmax＜1600cGy |
|  | Dmean＜1000cGy |
| Lens_L | D_max_＜600cGy |
| Lens_R | D_max_＜600cGy |
| OpticChiasm | D_max_＜3750cGy |
| OpticNrv_L | D_max_＜3750cGy |
| OpticNrv_R | D_max_＜3750cGy |
| Pituitary | D_max_＜3750cGy |
| Eye_L | D_max_＜3000cGy |
| Eye_R | D_max_＜3000cGy |
| Brainstem | D_max_＜3750cGy |

**TABLE S3** Simulated localization and image-guided scanning parameters.

| **Parameter item** | **Specific setting** |
| --- | --- |
| Scan range | From the cranial vertex to the cricothyroid membrane level |
| Scan mode | Helical scan |
| Tube voltage | 120 kVp |
| Tube current | 320 mAs |
| Slice collimation | 0.6 mm |
| Pitch | 0.6875 |
| Reconstruction algorithm | Soft tissue algorithm (H_SOFT_B) |
| Reconstruction slice thickness | 2.0 mm |
| Reconstruction interval | 2.0 mm |
| Field of view (FOV) | ~350 mm × 350 mm |
| Matrix | 512 × 512 |

**TABLE S4** Mini-Mental State Examination and Basic Activities of Daily Living for patients described in TABLE S1

| Patient | MMSE(score) | | | BADL(score) | | | Survival after RT(month) |
| --- | --- | --- | --- | --- | --- | --- | --- |
|  | Pre - RT | Post - RT | 3 months later | Pre - RT | Post - RT | 3 months later |  |
| 1 | 9 | 24 | / | 40 | 70 | / | 3 |
| 2 | 24 | / | / | 50 | / | / | 1 |
| 3 | 7 | 7 | / | 5 | 5 | / | 1 |
| 4 | 18 | 25 | / | 35 | 55 | / | 2 |
| 5 | 30 | 30 | / | 55 | 75 | / | 2 |
| 6 | 12 | 25 | 25 | 15 | 55 | 65 | Alive(＞13*) |
| 7 | 25 | 28 | 29 | 40 | 80 | 95 | Alive(＞14) |
| 8 | 12 | 20 | 19 | 25 | 85 | 65 | 6 |
| 9 | 21 | 25 | 27 | 40 | 85 | 100 | Alive(＞12) |
| 10 | 27 | 29 | 26 | 50 | 100 | 90 | Alive(＞10) |
| 11 | 29 | 29 | 29 | 60 | 95 | 95 | Alive(＞9) |

**Abbreviations: RT = radiotherapy**

*** Follow-up by August 26, 2024.**


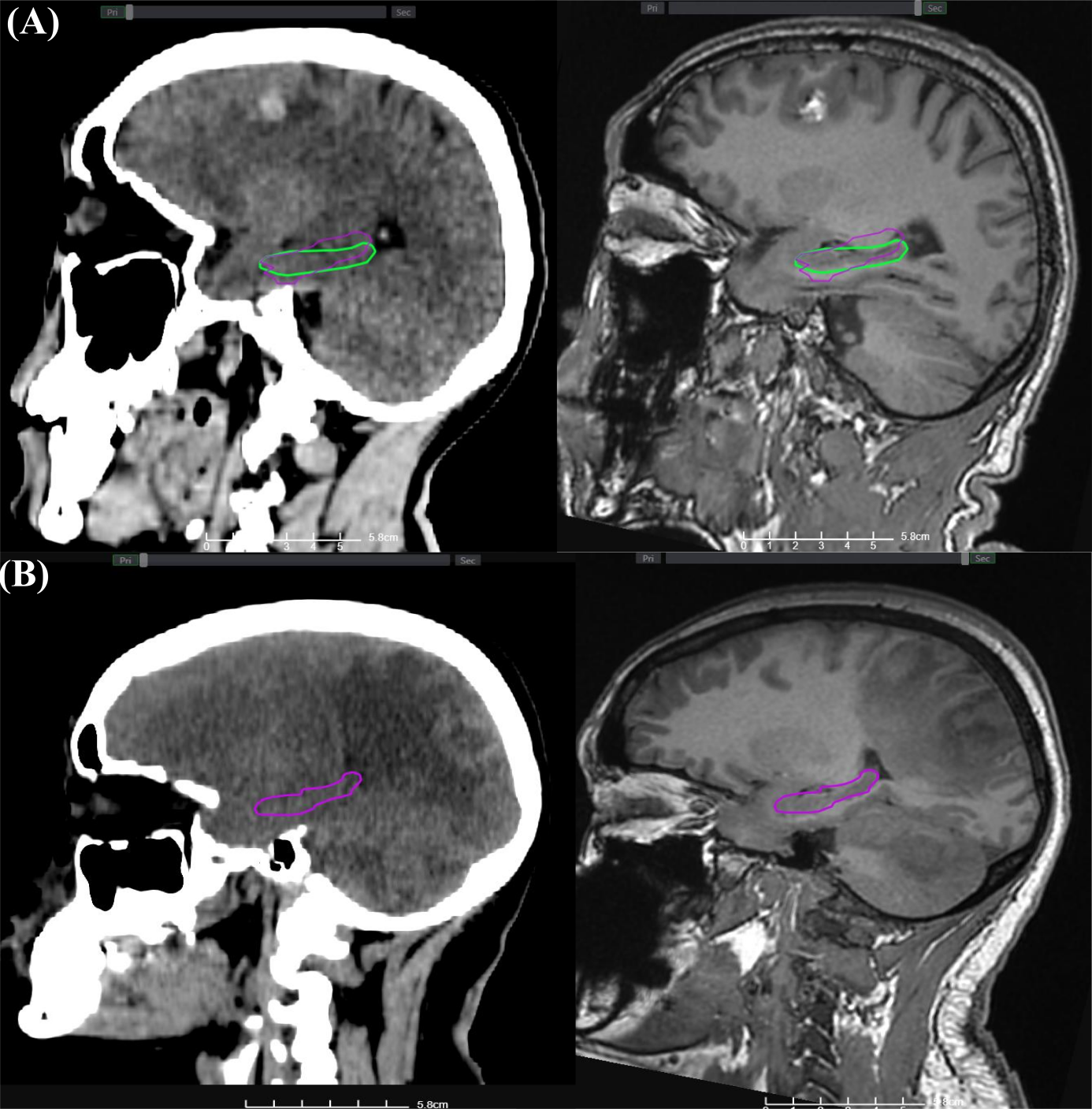


**FIGURE S3** Hippocampal Segmentation Based on CT Auto-segmentation and MRI Verification. (A) A representative case requiring substantial modification, (B) An auto-segmented case meeting the clinical standard. The purple contour represents the auto-segmentation result, while the green contour indicates the modified result.
